# Neoantigen-specific immunity in low mutation burden colorectal cancers of the consensus molecular subtype 4

**DOI:** 10.1186/s13073-019-0697-8

**Published:** 2019-12-30

**Authors:** Jitske van den Bulk, Els M. E. Verdegaal, Dina Ruano, Marieke E. Ijsselsteijn, Marten Visser, Ruud van der Breggen, Thomas Duhen, Manon van der Ploeg, Natasja L. de Vries, Jan Oosting, Koen C. M. J. Peeters, Andrew D. Weinberg, Arantza Farina-Sarasqueta, Sjoerd H. van der Burg, Noel F. C. C. de Miranda

**Affiliations:** 1 Pathology, LUMC, Postbus 9600, 2300 RC Leiden, The Netherlands; 2 Medical Oncology, Oncode Institute, LUMC, Leiden, The Netherlands; 3 Earle A. Chiles Institute, Portland, USA; 4 Immunohematology and Blood Transfusion, LUMC, Leiden, The Netherlands; 5 Surgery, LUMC, Leiden, The Netherlands; 6 AgonOx, Portland, USA

**Keywords:** Mismatch repair-proficient (MMR-p), Tumor-infiltrating lymphocytes, Transforming growth factor-beta, Cancer immunotherapy, Adoptive T cell transfer (ACT), Low mutation burden, Neoantigen, CMS

## Abstract

**Background:**

The efficacy of checkpoint blockade immunotherapies in colorectal cancer is currently restricted to a minority of patients diagnosed with mismatch repair-deficient tumors having high mutation burden. However, this observation does not exclude the existence of neoantigen-specific T cells in colorectal cancers with low mutation burden and the exploitation of their anti-cancer potential for immunotherapy. Therefore, we investigated whether autologous neoantigen-specific T cell responses could also be observed in patients diagnosed with mismatch repair-proficient colorectal cancers.

**Methods:**

Whole-exome and transcriptome sequencing were performed on cancer and normal tissues from seven colorectal cancer patients diagnosed with mismatch repair-proficient tumors to detect putative neoantigens. Corresponding neo-epitopes were synthesized and tested for recognition by *in vitro *expanded T cells that were isolated from tumor tissues (tumor-infiltrating lymphocytes) and from peripheral mononuclear blood cells stimulated with tumor material.

**Results:**

Neoantigen-specific T cell reactivity was detected to several neo-epitopes in the tumor-infiltrating lymphocytes of three patients while their respective cancers expressed 15, 21, and 30 non-synonymous variants. Cell sorting of tumor-infiltrating lymphocytes based on the co-expression of CD39 and CD103 pinpointed the presence of neoantigen-specific T cells in the CD39^+^CD103^+^ T cell subset. Strikingly, the tumors containing neoantigen-reactive TIL were classified as consensus molecular subtype 4 (CMS4), which is associated with TGF-β pathway activation and worse clinical outcome.

**Conclusions:**

We have detected neoantigen-targeted reactivity by autologous T cells in mismatch repair-proficient colorectal cancers of the CMS4 subtype. These findings warrant the development of specific immunotherapeutic strategies that selectively boost the activity of neoantigen-specific T cells and target the TGF-β pathway to reinforce T cell reactivity in this patient group.

## Background

Colorectal cancer (CRC) is the third most common cancer worldwide and was responsible for nearly 900,000 deaths in 2018 [1]. To improve cure rates for patients with advanced stage CRC, innovative treatment options are urgently needed. The recent advent of T cell checkpoint blockade-targeting immunotherapy has revolutionized the treatment of several cancers, but this therapeutic modality has only been effective in CRC patients diagnosed with mismatch repair-deficient (MMR-d) tumors [2–4]. MMR-d cancer cells fail to repair nucleotide substitutions as well as small nucleotide insertions and deletions that occur during DNA replication. Thereby, MMR-d tumors generally present with genomes carrying over 10 mutations per megabase, resulting in the expression of hundreds of proteins carrying non-synonymous mutations. Their immunogenic character and sensitivity to checkpoint blockade is considered to be largely derived from the recognition of somatically mutated antigens (neoantigens) by autologous T cells [5–8], in line with the strong association between mutation burden and clinical responses to checkpoint blockade in different types of solid cancers [3, 4, 8–11]. However, the majority of CRC (up to 80% of cases) comprise mismatch repair-proficient (MMR-p) tumors with low to moderate mutation burden and are currently not amenable to immunotherapeutic interventions. CRC can also be classified according to their transcriptional profiles into consensus molecular subtypes (CMS) that carry biological and clinical significance [12]. CMS1 is dominated by MMR-d CRC with strong immune infiltration, while CMS2 and CMS3 are characterized by Wnt pathway activation and metabolic dysregulation, respectively. Lastly, CMS4 is defined by a mesenchymal signature where the stromal compartment and TGF-β signaling play a major role. Of note, patients diagnosed with CMS4 CRC have worse survival than patients diagnosed with the other subtypes [13].

The activation of an effective anti-tumor immune response requires cancer antigens to be taken up and processed by antigen-presenting cells (APCs) which in turn present antigen-derived peptides to CD8^+^ and CD4^+^ T cells in complex with HLA class I and II molecules, respectively [14]. The molecular features of neoantigens and their affinity to the various intermediates of the antigen processing pathway determine whether they will be presented at the cell surface [15]. Therefore, the probability that a neoantigen is presented to a cognate T cell is reduced in cancers with low mutation burden, such as MMR-p CRC, thereby explaining why the clinical applicability of reactivating anti-cancer T cell responses has been mainly restricted to MMR-d CRC.

Nevertheless, the priming of neo-epitope-specific T cells in these cancers, despite their low mutation burden, would support the development of neoantigen-specific immunotherapeutic strategies, including neoantigen vaccination or adoptive transfer of neoantigen-specific T cells [16–18]. To address this possibility, we investigated the presence of neoantigen-specific T cell responses in tumor-infiltrating lymphocytes (TIL) and peripheral blood lymphocytes (PBL) of seven MMR-p CRC patients. In parallel, we characterized the immunophenotypes of these tumors by multispectral immunofluorescence imaging. Neoantigen-specific T cell reactivity could be detected in three out of seven MMR-p cases, all with a CMS4 transcriptional profile, which is associated with worse clinical prognosis [12]. This finding supports the design of specific immunotherapeutic strategies that target neoantigens in this patient group and suggests that an increased number of CRC patients could benefit from immunotherapeutic interventions.

## Methods

### Collection of patient material

**Fig. 1:**
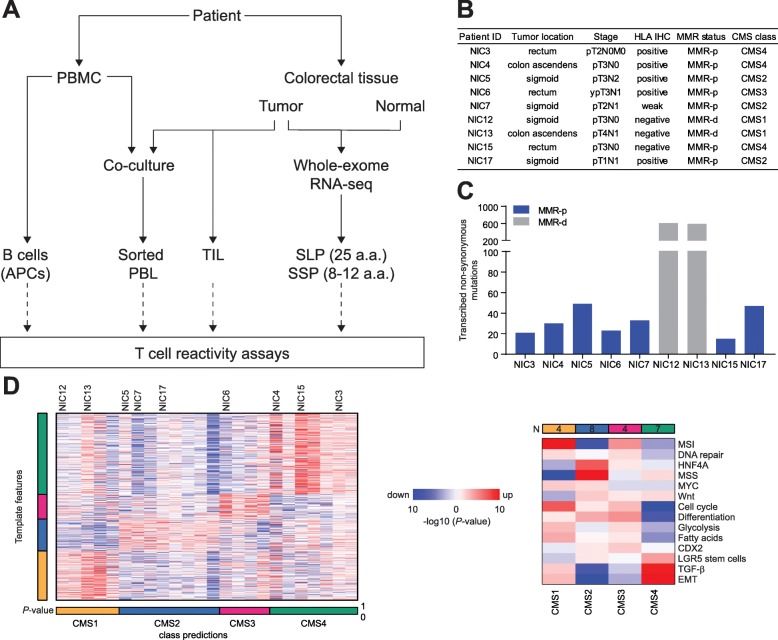
Neoantigen detection in low mutation burden CRC. **a** Schematic overview of the experimental design. **b** Patient characteristics including HLA class I phenotypes and MMR status of the tumors. **c** Total number of transcribed, non-synonymous mutations per patient. **d** Heatmaps showing the relative expression for template genes (left) and gene set (right) used to determine the consensus molecular subtypes of CRC samples. Color saturation indicates the statistical significance; red and blue indicate the direction of change. The samples analyzed included the tumors that were investigated for neoantigen reactivity and additional 15 CRC samples for which RNA sequencing was available in-house.

This study was approved by the Medical Ethical Committee of the Leiden University Medical Centre (protocol P15.282), and all patients provided informed consent. Methodological procedures as well as clinical stage, tumor location, and MMR status of the nine patients that underwent whole-exome and transcriptome sequencing are summarized in Fig. 1a, b. MMR status was determined initially through diagnostic procedures by making use of PMS2 and MSH6 immunodetection and was further confirmed by the observation of numerous nucleotide insertions and deletions by exome sequencing in the samples classified as MMR-d. Patient samples were anonymized and handled according to the medical ethical guidelines described in the Code of Conduct for Proper Secondary Use of Human Tissue of the Dutch Federation of Biomedical Scientific Societies. This research was conducted according to the recommendations outlined in the Helsinki Declaration.

Blood samples were obtained prior to surgery. Peripheral blood mononuclear cells (PBMC) were isolated from patients’ heparinized venous blood by Ficoll-Amidotrizoate (provided by the LUMC pharmacy) gradient centrifugation. Tumor material and respective normal colorectal samples were obtained immediately after surgery under supervision of a pathologist. A fraction of the tumor samples was snap-frozen; another part was cut into small fragments and digested using 1 mg/mL collagenase D (Roche, Basel, Switzerland) and 50 μg/mL DNAse I (Roche) in IMDM medium (Lonza BioWhittaker, Breda, The Netherlands) supplemented with 2 mM Glutamax (Thermo Fisher Scientific, Waltham, MA, USA), 20% Fetal Bovine Serum (Sigma-Aldrich, Saint Louis, MO, USA), 1% penicillin/streptomycin (Thermo Fisher Scientific), 1% Fungizone (Thermo Fisher Scientific), 0.1% Ciprofloxacin (provided by the LUMC pharmacy), and 0.1% Gentamicin (Sigma-Aldrich). Tissue fragments were incubated for 30 min at 37 °C interrupted by three mechanical dissociations on a gentleMACS Dissociator (Miltenyi Biotec, Bergisch Gladback, Germany) in gentleMACS C tubes (Miltenyi Biotec), and subsequently processed through a 70-μm strainer (Miltenyi Biotec). Single cell digests and remaining tumor fragments were cryopreserved for analysis and culturing at later stages. Additionally, 6–12 tumor fragments were directly employed for culturing of tumor-infiltrating lymphocytes (TIL).

### Whole-exome and RNA sequencing of tumor and corresponding normal tissue

Sequencing libraries were prepared from genomic DNA isolated from snap-frozen samples of tumor and corresponding normal colorectal tissue. NEBNext Ultra II DBA Library Prep kit for Illumina (New England Biolabs, Ipswich, MA, USA) and IDT xGEN Exome target kit (Integrated DNA Technologies, Leuven, Belgium) were used according to the manufacturer’s instructions for preparation of exome libraries. NEBNext Ultra Directional RNA Library Prep kit for Illumina (New England Biolabs) was used according to the manufacturer’s instructions to generate RNA sequencing libraries. rRNA was depleted from total RNA using the NEBNext rRNA depletion kit (New England Biolabs). The obtained paired-end, 150-bp libraries were sequenced at GenomeScan (Leiden, The Netherlands) on a HiSeq4000 Illumina, aimed at generating 11-Gb and 15-Gb datasets per sample for exome and transcriptome libraries, respectively.

For exome sequencing, reads were mapped against the human reference genome (hg38) using the Burrows-Wheeler Aligner 3 algorithm (BWA-mem version 0.7.15) [19]. Duplicate reads were removed using Picard Tools [20]. Genome Analysis Toolkit 7 (GATK version 3.8; Broad Institute, Cambridge, MA, USA) was used for base quality recalibration. OptiType was used to genotype HLA class I alleles from RNA and exome sequencing data (Additional file 1: Table S1) [21]. Subsequently, variant calling was done using a combination of three software tools, muTect 2, varScan 2, and Strelka [22–24]. The resulting .vcf files were then combined into a single file using GATK CombineVariants [25]. Integrative Genomics Viewer (IGV, Broad Institute) was used for visual inspection of the variants [26–28]. Variants were functionally annotated using the Ensembl Variant Effect Predictor (VEP) [29]. With exception of synonymous substitutions, all other coding variants were further investigated if at least one read displaying a mutation was present in the RNA sequencing data. To this purpose, RNA sequencing reads were first mapped against the same hg38 genome build using gsnap [30], followed by read count at variant positions using the samtools mpileup tool. Allele frequencies at DNA level were extracted from the .vcf files and an mpileup file was generated for all mutated sites to inform on the number of variant-supporting reads at RNA level. Purity estimates of the tumor content were determined using Sequenza [31].

Twenty-five-mer peptide sequences were generated for all the identified variants. In case of frameshifts and stop loss mutations, several peptides were generated which overlapped for at least half of the sequence. Furthermore, affinity prediction of short peptides (8–12 mers) to the patients’ HLA alleles was performed using NetMHC 4.0 and NetMHCpan 4.0, defining top-ranked strong and weak binders [32–34]. All long peptides corresponding to mutations as well as short peptides classified as strong binders (0.5% top rank) were synthesized by the Cell and Chemical Biology department at the Leiden University Medical Center. In addition, for those variants without any strong binders, the short peptide with highest binding affinity to any HLA class I allele was also tested (Additional file 2: Table S2).

### CMS classification and immune signatures

CMScaller R package was used for both Consensus Molecular Subtyping (CMS) and Gene Set Analysis (GSA) on the colorectal cancer TCGA dataset and our own cohort (Leiden cohort) [35]. For the TCGA dataset, HTSeq counts from 449 primary tumors (one per sample) were downloaded from the Genomic Data Commons portal (https://portal.gdc.cancer.gov/). For the Leiden cohort, gene expression counts were obtained using HTseq-count [36]. GSA was performed on both datasets for the 14 transcriptional signatures described by Eide and colleagues [35] and an immune-regulatory gene set that was designed based on the Molecular Signatures Database IMMUNE_RESPONSE gene set (http://software.broadinstitute.org/gsea/msigdb/cards/IMMUNE_RESPONSE, Additional file 3: Table S3). Differential gene expression between the CMS2/3 groups and the CMS4 samples was investigated on the TCGA cohort by employing the Limma-Voom package after TMM normalization of the HTseq counts with the edgeR package [37, 38]. Genes were considered differentially expressed if they had a log_2_ fold-change below or above − 1 and 1, respectively, and an adjusted *P* value lower than 0.05. The immune-regulatory genes that were shown to be differentially expressed in the TCGA dataset were further investigated in the Leiden cohort.

### T cell expansion and B cell immortalization

TIL expansion was performed by culturing tumor fragments in a 24-well plate with T cell medium (IMDM (Lonza BioWhittaker)), supplemented with 7.5% heat-inactivated pooled human serum (Sanquin, Amsterdam, The Netherlands); penicillin (100 IU/mL), streptomycin (100 μg/mL), and l-glutamine (4 mM) (Lonza Biowhittaker); and rIL-2 (1000 IU/mL, Aldesleukin, Novartis). After 14–21 days of culturing, TIL were harvested and cryopreserved for later use. Rapid expansion of TIL was performed to increase the number of T cells available for reactivity assays. The expansion was induced by culturing the TIL with rIL-2 (3000 IU/mL), OKT3 (Miltenyi Biotec, 30 ng/mL), and irradiated (40 Gy) feeder cells (100–200 fold excess) for 4–5 days. Feeder cells were PBMC, derived from healthy donor blood provided by Sanquin (The Netherlands), and isolated by density centrifugation with Ficoll, as described for the patients’ blood. Subsequently, culturing was continued up to 2 weeks in T cell medium with rIL-2 (3000 IU/mL) [18]. Phenotyping of the expanded TIL was performed by flow cytometric analysis of CD4, CD8, FoxP3, CD45RA, CD45RO, CD39, CD103, and PD-1 expression (Additional file 4: Table S4A). Cells were incubated for 45 min with the cell surface antibodies and a live/dead marker. Subsequently, cells were treated with the Transcription Factor Staining Buffer Set (eBioscience, San Diego, CA, USA) to prepare cells for FoxP3 detection. Samples were measured on an LSRFortessa machine (BD, Franklin Lakes, NJ, USA), and the data was analyzed using FlowJo software v10.2 (BD).

Epstein-Barr virus–transformed lymphoblastoid B cell lines (EBV-LCL) were used as antigen-presenting cells (APCs). Their immortalization was induced by incubating patients’ PBMC with supernatant of the marmoset B cell line containing infectious particles of EBV strain B95-8 for 1 h at 37 °C. Culture medium consisted of RPMI-1640, supplemented with 5 μg/mL PHA (Thermo Fisher Scientific), 10% FCS, l-glutamine (4 mM), penicillin (100 μg/mL), and streptomycin (100 μg/mL). Cells were refreshed every 5–6 days with B cell medium and cultured for 3 weeks before being used as APCs.

Tumor-reactive lymphocytes from peripheral blood were generated by co-culture of PBMC with lethally irradiated (100 Gy) tumor fragments in T cell medium and subsequent isolation of PD-1-positive cells [39]. Cells were harvested and stained with PE-labeled anti-PD-1 antibodies (BD Biosciences). Next, MACS cell sorting was performed by use of magnetic anti-PE beads (Miltenyi Biotec) and MS columns (Miltenyi Biotec). PD-1-positive cells as well as flow-through were expanded as described above for the TIL cultures. Culture medium containing rIL-2 was refreshed on alternate days. Cells were cryopreserved after a culturing period of 2 weeks.

CD39^+^CD103^+^ CD8^+^ T cell fractions were sorted and cultured as described previously [40]. In short, single-cell suspensions derived from tumor digests were stained to perform a flow cytometric cell sort of the cell types of interest based on phenotypic markers using the following antibodies: CD45 FITC (BioLegend, San Diego, CA, USA; 2D1), CD4 BV785 (BioLegend), CD8 BV510 (BioLegend, RPA-T8), CD45RA APC-780 (eBioscience, San Diego, CA, USA; HI100), CCR7 PE/Dazzle 594 (BioLegend, G0443H7), CD39 APC (eBioscience, eBioA1), and CD103 PE (eBioscience, B-Ly). The sorted cells were cultured in RPMI-1640, supplemented with 2 mM glutamine, 1% non-essential amino acids, 1% sodium pyruvate, penicillin (50 IU/mL), streptomycin (50 μg/mL) and 10% fetal bovine serum (Hyclone, South Logan, UT, USA). T cells were stimulated with 1 μg/mL PHA (Remel) in the presence of irradiated (40 Gy) allogeneic feeder cells (2 × 10^5^ cells/well) and 10 ng/mL IL-15 (BioLegend) in a 96-well round-bottom plate. The T cells were maintained in complete medium containing IL-15 until cryopreservation.

### T cell reactivity

Reactivity of T cells to tumor material and/or neoantigens was investigated by a co-culture reactivity assay. In order to screen for neoantigen reactivity, autologous EBV-LCL were placed in overnight co-culture with 20 μg/mL of synthetic long peptides (SLP). Synthetic short peptides (SSP) were directly added at a concentration of 2 μg/mL to T cells, without addition of EBV-LCL. Fifteen-thousand T cells were tested per condition including overnight co-cultures with irradiated (60 Gy) tumor material, SSP, or 30.000 EBV-LCL loaded with SLP. Unloaded EBV-LCL or medium supplemented with and without DMSO corresponding to the peptide solution, served as negative controls. *Staphylococcus aureus* enterotoxin B (SEB; 0.5 μg/mL; Sigma-Aldrich) was used as positive control. T cell reactivity was primarily determined by IFN-γ secretion in the supernatant, measured by ELISA (Sanquin or Mabtech, Stockholm, Sweden). In addition, CD137 expression on T cells, measured by flow cytometric analysis with a panel targeting CD3, CD4, CD8, CD137, and a live/dead marker, was used as an activation read-out. Antibody details and the settings of the LSRFortessa machine (BD, Franklin Lakes, NJ, USA) can be found in Additional file 4: Table S4B. To detect reactivity against tumor material, granzyme B secretion was also assessed by ELISA (Mabtech) and T cells were harvested for RNA isolation with Nucleospin RNA XS kit (Macherey Nagel, Düren, Germany), according to the manufacturer’s instructions. Gene expression was measured by qPCR with the SsoFast Evagreen Supermix (Bio-Rad, Hercules, CA, USA) and the following primer pairs: *IFNG* Fw ACACTCTTTTGGATGCTCTGGT; *IFNG* Rv TTGGAAAGAGGAGAGTGACAGAA; *GZMB* Fw GATGCAGGGGAGATCATCGG; *GZMB* Rv CCGCACCTCTTCAGAGACTT; *TNFRSF9* AGAGAGGTCGGCTGGAGATG; and *TNSRSF9* Rv CCCTGGACAAACTGTTCTTTGGA.

### Immunohistochemistry and immunofluorescence

Formalin-fixed, paraffin-embedded tissue slices of 4 μm were cut on glass slides for immunohistochemical or immunofluorescence detection. Tissue sections were deparaffinized by xylene and rehydrated by decreasing concentrations of alcohol solutions. Endogenous peroxidase was blocked with 0.3% hydrogen peroxide in methanol solution for 20 min. Pre-treatment of the sections included heat-induced antigen retrieval in pH 6.0 citrate buffer (10 mM, not used for β2-microglobulin detection). Primary antibodies were diluted in PBS with 1% BSA and incubated overnight. Three antibodies against the heavy and light chains of the HLA class I molecules (HCA2 1:3200 (Nordic MUbio, Susteren, The Netherlands), HC10 1:3200 (Nordic MUbio), and β2-microglobulin (B2M) 1:100 (Dako, Carpinteria, CA, USA)) were used for immunohistochemical detection. The secondary antibody, a polymeric HRP-linker antibody conjugate (Immunologic, Duiven, The Netherlands), was incubated for 1 h, followed by development using DAB+chromogen (Dako) for 5 min. Counterstaining was performed with hematoxylin for 30 s. Finally, sections were dehydrated by increasing amounts of alcohol followed by xylene. Slides were mounted using Pertex. Expression of HLA class I was assessed in every tumor section using the scoring system: positive, negative, or weak [41]. Scoring took place against the internal control, provided by stromal and immune cells.

For T cell infiltrate analysis, additional tissue sections were used for immunofluorescence detection of Keratin, CD3, CD8, and FoxP3 as previously reported [42]. In short, pH 6.0 citrate buffer was used for heat-induced antigen retrieval. Superblock buffer (Thermo Fisher Scientific) was applied, and subsequently, all primary antibodies that were detected indirectly by isotype-specific fluorescent-labeled antibodies were incubated overnight (CD8 and FoxP3). Then, the secondary antibodies were applied, followed by incubation with the directly conjugated antibodies (CD3-AF594 and Keratin-AF488). Finally, a nuclear counterstain was performed with 1 μM DAPI. Analysis was performed using the Vectra 3.0 Automated Quantitative Pathology Imaging System (Perkin Elmer, Waltham, MA, USA) which captured × 20 magnification images. The software was trained to segment tissues into tumor, stroma, and “no tissue” areas, followed by cellular segmentation. Subsequently, the software assigned phenotypes to all cells according to the expression of the markers employed. Cell counts were normalized by tissue area (number of cells/mm^2^).

### Statistics

Student’s *t* test was applied to test differential reactivity to wild type and mutant peptides with Bonferroni’s correction for multiple testing. One-way ANOVA was employed for detecting differences in granzyme B secretion upon co-culture of TIL with tumor fragments. These tests and graphical representation were performed with Graphpad Prism 8.0.1.

## Results

### The neoantigen landscape of mismatch repair-proficient colorectal cancers

We determined the mutational profiles of seven mismatch repair-proficient (MMR-p) and two mismatch repair-deficient (MMR-d) CRC by whole-exome and transcriptome sequencing of cancer tissues and respective normal colonic mucosa (Fig. 1a, b). All non-synonymous (i.e., missense mutations, nucleotide insertions, and deletions leading to frameshift and non-frameshift mutations, stop loss mutations, and splicing mutations) somatic mutations were considered as potential neoantigens. We identified 15 to 49 transcribed, non-synonymous somatic mutations in MMR-p CRC (Fig. 1c). In comparison, the same approach led to the discovery of approximately 20 times more mutations in the MMR-d cancers. Patient-specific HLA class I alleles were typed from the transcriptome and whole-exome sequencing data generated from tumor and healthy tissues which showed full concordance (Additional file 1: Table S1).

HLA class I expression in cancer tissues was investigated by immunohistochemistry with antibodies against the HLA class I heavy-chain. Membranous HLA class I expression was retained in the majority of MMR-p cancers while lost in NIC15 (MMR-p tumor) and both MMR-d samples (Fig. 1b). This indicates that the antigen processing machinery is still operational in most MMR-p tumors. No genetic basis for loss of HLA class I expression in sample NIC15 could be found after analysis of the exome and RNA sequencing data while frameshift mutations in the *HLA-A* (NIC12 and NIC13) and *CANX* (NIC13) genes were discovered in the MMR-d samples. Transcriptome analysis of the NIC samples together with an additional 15 CRC samples (Leiden cohort) was used to classify the tumors according to the consensus molecular subtypes of CRC [35]. In accordance with their MMR-d status, NIC12 and NIC13 were classified into the CMS1 subtype, while the MMR-p samples were classified as belonging to the CMS2, 3, or 4 subtypes (Fig. 1d).

### Detection of neoantigen-specific T cell responses in low mutation burden CRC

Neoantigen recognition in the MMR-p cancers was tested by stimulation of the different T cell cultures with SSP and EBV-LCL loaded with SLP (Fig. 1a). T cell reactivity was measured based on IFN-γ production as detected by ELISA, and expression of the activation marker CD137, assessed by flow cytometry.

**Fig. 2:**
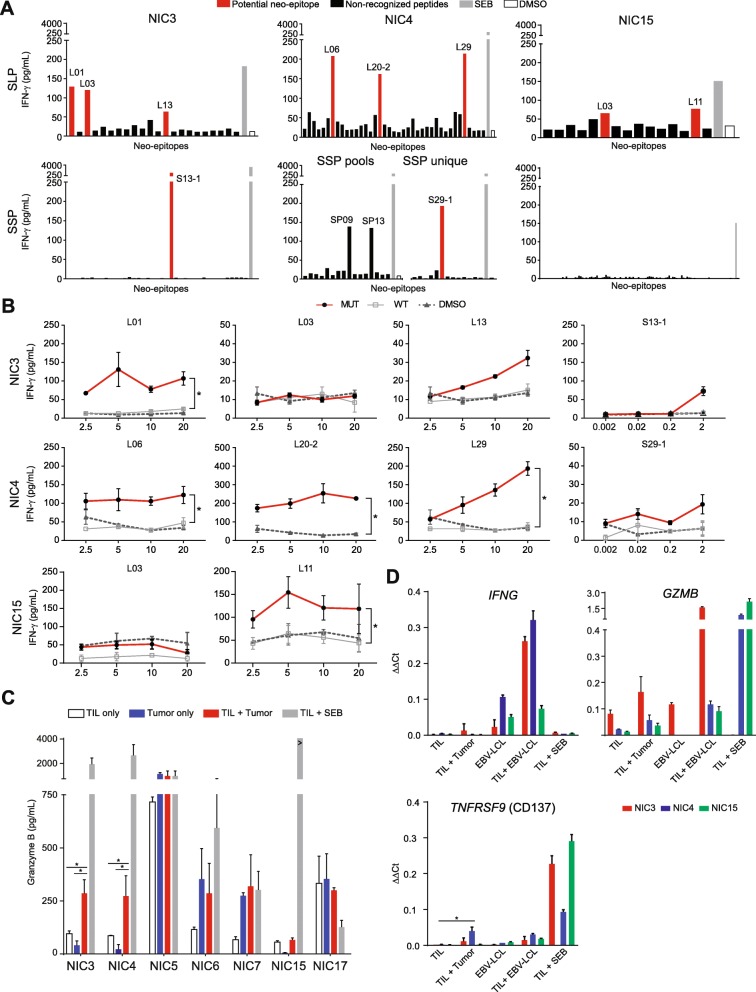
Neoantigen-specific T cell reactivity in MMR-p CRC. **a** IFN-γ production of expanded TIL in response to synthetic long peptides (SLP) and synthetic short peptides (SSP), potential neo-epitopes in red and non-recognized peptides in black. SEB (gray) and DMSO (white) were taken along as positive and negative controls, respectively. Peptide IDs are included for neo-epitope responses that were judged positive and selected for validation. SSP and SLP with the same ID number correspond to the same mutation per patient. **b** IFN-γ production of TIL upon co-culture with mutant (red) and corresponding wild type (gray) peptides, and a DMSO control (dashed), at different peptide concentrations. The mean ± standard deviation of the biological duplicates in the same experiment are depicted. An asterisk indicates a significant difference (*α* = 0.0026) between wild type and mutant peptides. **c** Granzyme B production by TIL upon stimulation with autologous tumor fragments (red). TIL only (white) and tumor only (blue) conditions were taken along as negative controls, and SEB (gray) as positive control. Differential production between TIL + tumor and TIL or tumor only is analyzed by ANOVA; the asterisks indicate significant differences. **d** Gene expression measured by qPCR upon co-culture of different target/effector combinations of NIC3 (red), NIC4 (blue), and NIC15 (green). Differential gene expression upon co-culture with wild type and mutant peptides is indicated with an asterisk.

An initial screening revealed potential neoantigen-reactivity in six out of the seven MMR-p CRC in both TIL- and PBL-derived T cell cultures (Fig. 2a; Additional files 5 and 6: Figure S1 and Figure S2). High IFN-γ production was observed when PBL-derived T cells were co-cultured with EBV-LCL in all samples, except NIC6, irrespective of the SLP loading. A similar observation was done with the TIL product of NIC5 and NIC17, suggesting the presence of EBV-reactive cells in these T cell products. Potential hits identified in the previous screen were validated with HPLC-purified, wild type, and mutant versions of the putative neoantigen sequences. A *bona fide*, neoantigen-specific T cell response was defined when T cells specifically reacted against the mutant peptide. Neoantigen-specific T cell reactivity was observed in the samples derived from patients NIC3, NIC4, and NIC15 (Fig. 2b; Additional file 7: Figure S3). For NIC3, T cell reactivity was confirmed against two SLP representing the mutations *PARVA* c. 328C>G (p.P110R, peptide L01) and *G3BP1* c. 244G>A (p.A82T, peptide L13) and an SSP (peptide S13-1) corresponding to the latter variant (Fig. 2b, Table 1). In NIC4, T cell responses were directed towards SLP corresponding to three different mutations: *ACTR10* c.638G>A (p.R213H, peptide L06), *RAE1* c.1106A>G (p.X369W, peptide L20-2), and *PDP1* c.1024C>T (p.R342W, peptide L29) (Fig. 2b, Table 1). In NIC15, T cell activity was detected towards a SLP representing the c.1054C>A (p.V352F) mutation in *QRICH1* (Fig. 2b, Table 1). The targeted genes lack any apparent involvement in CRC oncogenesis, but importantly, they were present among the dominant tumor clones as determined by the mutated allele frequency and estimated tumor cell fractions (Table 1; Additional file 2: Table S2). Furthermore, the RNA expression levels of neoantigen-encoding genes were comparable to the ones of genes encoding the remaining non-recognized mutations (Additional file 8: Figure S4A). In these patients, 20 (NIC3), 35 (NIC4), and 15 (NIC15) putative neoantigens had been identified by sequencing which translates to a neoantigen detection rate of 10%, 9%, and 6.7%, respectively. No neoantigen reactivity was observed in blood-derived T cells (Additional file 7: Figure S3), although the analysis was likely hampered by EBV-directed background reactivity as a result of EBV-transformed B cells being employed as APCs. Furthermore, the specific selection of PD-1^hi^ subsets might have been more successful for pre-selection of tumor-specific T cells [43, 44].

**Table 1:** Patient’s neo-epitopes to which T cell reactivity was detected.

Patient	% Tumor	# Mut	# SLP	# SSP	Genes	Mut cDNA	Mut a.a.	% Mut (WES)	Peptide	Peptide ID
NIC3	21	21	24	47	*PARVA*	c.328C>G	p.P110R	11	NLPLSPIPFELDREDTMLEENEVRT	L01
					*G3BP1*	c.244G>A	p.A82T	11	NCHTKIRHVDAHTTLNDGVVVQVMG	L13
					*G3BP1*	c.244G>A	p.A82T	11	IRHVDAHTTL	S13-1
NIC4	48	30	39	46	*ACTR10*	c.638G>A	p.R213H	15	SVPEGVLEDIKAHTCFVSDLKRGLK	L06
					*RAE1*	c.1106A>G	p.X369W	13	WWLETLAQPELFLSTLPHLCTNLGP	L20-2
					*PDP1*	c.1024C>T	p.R342W	45	PKSEAKSVVKQDWLLGLLMPFRAFG	L29
					*PDP1*	c.1024C>T	p.R342W	45	SEAKSVVKQDW	S29-1
					*PDP1*	c.1024C>T	p.R342W	45	SEAKSVVKQDWL	S29-2
NIC5	72	49	71	94	–	–	–	–	–	–
NIC6	79	23	24	32	–	–	–	–	–	–
NIC7	78	33	44	70	–	–	–	–	–	–
NIC15	43	15	15	108	*QRICH1*	c.1054C>A	p.V352F	14	VHVSGSPTALAAFKLEDDKEKMVGT	L11
NIC17	51	45	47	60	–	–	–	–	–	–

*% Tumor* tumor purity, *Mut* mutation, *SLP* synthetic long peptides, *SSP* synthetic short peptides, *WES* reads in whole-exome sequencing.

To investigate if the observed T cell responses were genuinely patient-specific, the TIL of NIC3 and NIC4 were stimulated with the putative neoantigen peptide pools from other patients (Additional file 9: Figure S5). No cross-reactivity was detected, emphasizing the patient-specific nature of the detected T cell responses.

### Tumor-directed T cell reactivity in MMR-p CRC

TIL were co-cultured with small, irradiated tumor fragments in order to assess whether tumor-directed T cell activity could be detected in the same samples where neoantigen-specific T cells were identified. Initially, tumor-reactivity was assessed in a similar manner to the neo-epitope screening and showed that the TIL cultures established from patient NIC4 produced IFN-γ upon stimulation with autologous cancer tissue. Furthermore, they also displayed increased CD137 expression in approximately 5% of CD8^+^ T cells (Additional file 8: Figure S4B, S4C; adjusted for negative control) indicating that tumor reactivity was restricted to a minority of TIL in this sample. Recently, other groups have reported discordance between IFN-γ production and CD137 expression in similar assays with CRC tissues, despite the true nature of neoantigen-specific reactivity [45]. To address potential issues related to the sensitivity of this approach, an additional strategy was employed to screen all samples by measuring granzyme B release in the supernatant of the co-cultures followed by gene expression analysis of TIL [46]. Granzyme B release was found to be increased compared to the negative controls in both NIC3 and NIC4 when TIL were co-cultured with tumor material (Fig. 2c). The same was not observed upon co-culture of NIC15 TIL with tumor material which may be explained by the fact that this tumor had lost HLA class I expression (Fig. 1b). In the same experimental setting, RNA was isolated from the different co-cultures and the expression levels of the *IFNG*, *GZMB* (granzyme B), and *TNFRSF9* (CD137) were assessed (Fig. 2d). While generally supportive of tumor-directed reactivity, it is striking that these genes behave differently as readouts depending on the sample but also on the type of stimuli, thus highlighting the need to redefine comprehensive and sensitive approaches for the identification of cancer-reactive T cells in CRC.

### CD39 and CD103 identify neoantigen-reactive CD8^+^ T cells

**Fig. 3:**
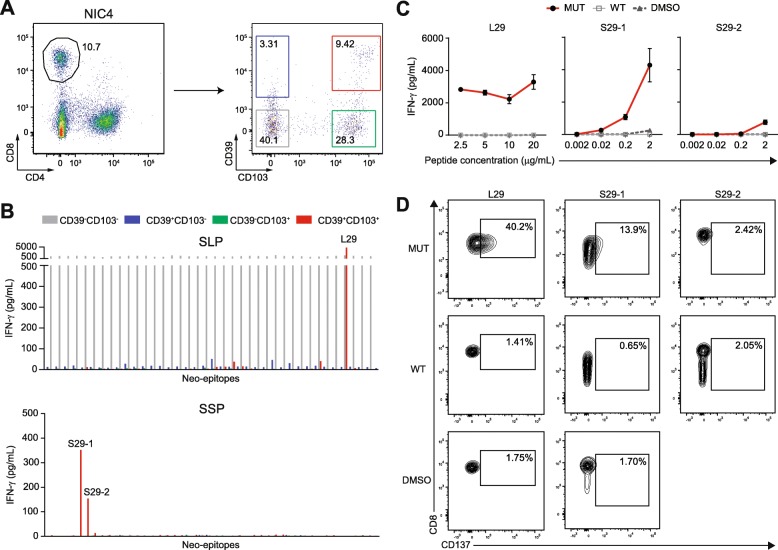
Neoantigen-reactivity is contained within CD39^+^CD103^+^ CD8^+^ T cell subsets. **a** Flow cytometric sorting procedure adopted for the isolation of CD8^+^ T cell subsets according to CD39 and CD103 expression. Numbers within the gates represent the percentage of CD8^+^ cells contained in each subset. **b** Neoantigen-specific responses of the different T cell subsets upon co-culture with neo-epitopes. Peptide numbers are included for responses that were determined to be positive, and were taken along in the validation experiment. **c** IFN-γ production of the CD39^+^CD103^+^ CD8^+^ T cells upon co-culture with mutant (black) and corresponding wild type (gray) peptides, and a DMSO control (dashed), at different peptide concentrations. The mean ± standard deviation of the biological duplicates in the same experiment are depicted. **d** Flow cytometric analysis of the percentage of CD137^+^ T cells, depicted in the gates, within the CD8^+^ population of the expanded TIL upon co-culture with the mutant or wild type peptide, or DMSO control.

Co-expression of CD39, an ectonucleotidase, and CD103, an integrin that pinpoints tissue-resident T cells, have been proposed to discriminate tumor-infiltrating, cancer-reactive CD8^+^ T cells [40]. We investigated whether neoantigen reactivity in MMR-p CRC was also compartmentalized into specific CD8^+^ T cell subsets defined by the aforementioned markers. To this end, CD8^+^ TIL from patient NIC4 were sorted by flow cytometry into double-negative, single-positive, and double-positive subsets according to CD39 and CD103 expression (Fig. 3a). Subsequently, these populations were expanded and tested for neoantigen reactivity towards all the mutant peptides of NIC4. Neoantigen-specific responses were specifically observed in the CD39^+^CD103^+^ CD8^+^ T cell subset. T cell activation was detected against the L29, S29-1, and S29-2 peptides (Fig. 3b), all derived from the *PDP1* c.1024C>T mutation that was shown to be recognized by T cells in the bulk TIL product (Table 1). This observation could be reproduced using HPLC-purified peptides harboring the neoantigen sequence, and its corresponding wild type sequence which did not elicit T cell activation (Fig. 3c). Approximately 40% of CD39^+^CD103^+^ CD8^+^ T cells expressed CD137 after being exposed to the L29 peptide, as opposed to 1.41% when using the wild type peptide (Fig. 3d). For S29-1 and S29-2, CD137 expression was found in 13.9% and 2.42% of CD39^+^CD103^+^ CD8^+^ T cells, respectively, compared to only 0.65% and 2.05% upon stimulation with the corresponding wild type peptide.

We did not observe reactivity against *ACTR10* c.638G>A (p.R213H) or *RAE1* c.1106A>G (p.X369W) in the sorted T cell fractions, which could possibly be explained by the fact that those responses were mediated by CD4^+^ T cells. In agreement, no reactivity was detected against SSP derived from the same mutations.

As previously reported, T cell reactivity directed to EBV-LCL was confined to the CD39^−^CD103^−^ CD8^+^ T cell subset [40, 47]. In this subset, IFN-γ production was detected against all SLP-loaded and unloaded EBV-LCL (Fig. 3b). This suggests that the sorting of specific T cell subsets prior to T cell expansion and T cell reactivity assays can enrich the number of tumor-specific T cells and facilitate the discovery of neoantigen-reactive T cells.

Additional single cell digests were not available for NIC3 and NIC15, and therefore, the compartmentalization of neoantigen reactivity within specific CD8^+^ T cell subsets could not be investigated in these samples.

### T cell reactivity correlates with CMS subtype and immune cell infiltration patterns

**Fig. 4:**
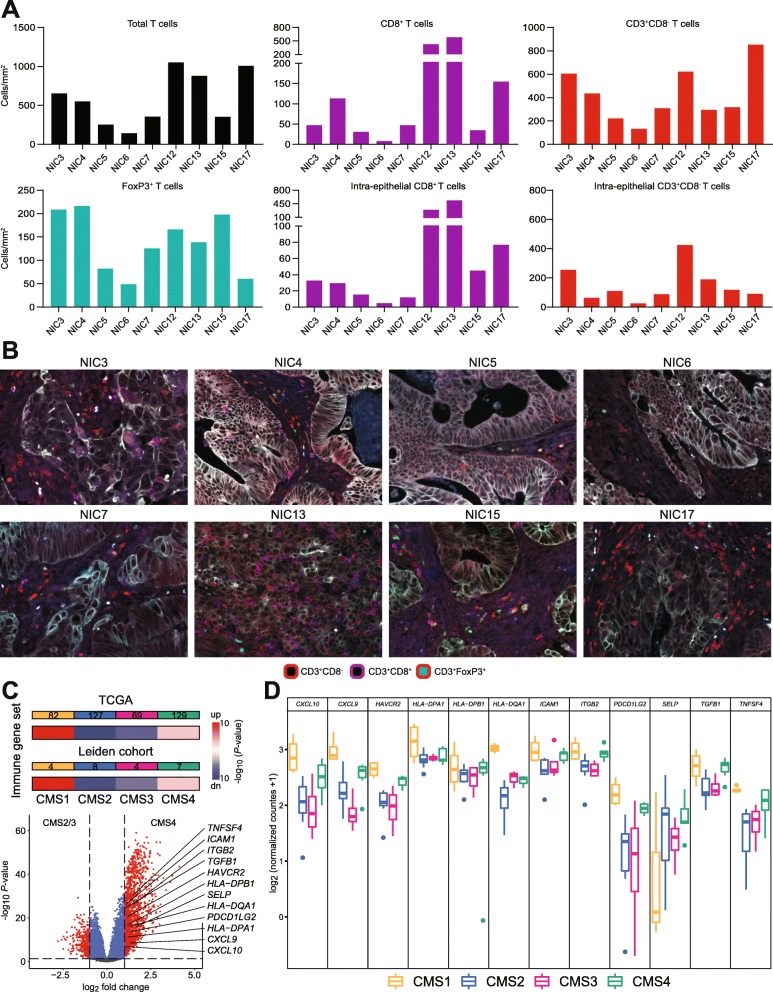
Immune infiltration and differentially expressed genes between NIC samples and CMS subtypes. **a** Quantitative analysis of immune cell infiltration by multispectral fluorescent imaging. The number of cells was counted per square millimeter of tissue (total) and epithelium (intra-epithelial). **b** Representative tissue sections demonstrating variable infiltration of immune cells in MMR-p (NIC3–7) and MMR-d tumors (NIC13). **c** Heatmaps showing the relative expression of immune regulatory genes for the CRC TCGA dataset and the Leiden cohort. Color saturation indicates the statistical significance; red and blue indicate the direction of change. Volcano plot shows differentially expressed genes between CMS2/3 (left) and CMS4 (right) samples. Statistically significant expressed genes from the immune gene set are depicted. **d** Box plot representing the gene expression per CMS subtype in the Leiden cohort of the differentially expressed immune genes determined in **c.**

All CRC in which neoantigen-directed T cell reactivity was detected (NIC3, NIC4, and NIC15) were classified as CMS4 according to their transcriptional profile, characterized by a strong mesenchymal signature associated with TGF-β pathway activation. The success rate of initial TIL culture and expansion or the phenotypical constitution of TIL samples do not indicate an increased likelihood of encountering neoantigen-specific T cell responses in the CMS4 subtype (Additional file 10: Table S5). To investigate differences in the quality and quantity of T cell infiltration in the samples screened for neoantigen reactivity, we performed multispectral fluorescence imaging (Fig. 4a, b). As expected, the highest number of T cells (total and CD8^+^ T cells) was found in the MMR-d samples NIC12 and NIC13. Interestingly, the samples with neoantigen reactivity displayed a high number of total T cells and intra-epithelial CD8^+^ T cells, compared to the other MMR-p samples. Strikingly, the density of FoxP3^+^ T cells in NIC3, NIC4, and NIC15 was higher than in any other sample. This observation is in line with the dominant role that TGF-β plays in these tumors as this growth factor is known to support the differentiation of regulatory T cells.

To determine whether CMS4 tumors displayed additional immune features that distinguish them from other MMR-p CRC, we investigated the expression of 78 immune-related genes (Additional file 3: Table S3) across CMS subtypes in the TCGA CRC dataset. Interestingly, an overall analysis placed the CMS4 group in between the CMS1 and CMS2/3 subtypes suggesting that immune features are more prominent in CMS4 tumors as compared to other MMR-p CRC. Twelve genes were determined to be upregulated in the CSM4 subtype when compared to the CMS2/3 group, including *TGFB1*, in line with the most prominent biological feature of the former subtype. In addition, genes encoding important molecules involved in immune cell trafficking (*CXCL9* and *CXCL10*) and cellular adhesion (*ICAM1*/*CD54*, *ITGB2*/*CD18*, and *SELP*), HLA class II genes, the T cell checkpoint gene *HAVCR2* (TIM-3), *TNFSF4* (OX40L), and *PDCD1LG2* (PD-L2) were all shown to be upregulated in the CMS4 subtype in comparison to the CMS2/3 group (Fig. 4c). Most of these genes were also shown to have increased expression in the CMS4 samples of the Leiden cohort in comparison to the CMS2/3 samples, albeit the lower number of samples (Fig. 4d). The expression of the CXCL9 and CXCL10 chemokines, together with HLA class II, OX40L, and PD-L2 are suggestive of the presence of antigen-presenting cells in the microenvironment while TIM-3 expression may reflect an activated/dysfunctional phenotype of tumor-infiltrating T cells. Of note, the expression of TIM-3, OX40 ligand, and PD-L2 were previously shown to be stimulated by TGF-β [48–50]. Altogether, we have found evidence that immune-related gene expression signatures are able to distinguish CRC of the CMS4 subtype from other MMR-p CRC.

## Discussion

The success of checkpoint blockade immunotherapies in patients diagnosed with cancers with high mutation burden [3, 4, 8–11] may emphasize the notion that tumors presenting few mutations are not amenable to immunotherapeutic strategies [3]. Here, we demonstrated that neoantigen-directed T cell responses occur naturally in CRC with low mutation burden. Specifically, we have detected responses against more than one neoantigen in three CRC cases that carried less than 50 transcribed, non-synonymous mutations. Interestingly, these cases belonged to the CMS4 molecular subtype, associated with a TGF-β-driven transcriptional signature and worse clinical outcome [12, 13]. Although these results are derived from a small cohort and thus do not exclude the possibility to detect neoantigen-specific responses in CMS2 and CMS3, it proposes TGF-β as an interesting therapeutic target to augment immune responses in patients diagnosed with CMS4 cancers. TGF-β itself might be responsible for keeping the anti-tumor activity of neoantigen-specific T cells at bay in those patients. TGF-β is known to promote the differentiation of CD4^+^ T cells into regulatory T cells (Tregs) [51], which is in line with the higher number of CD3^+^FoxP3^+^ cells that were observed in the CMS4 cases infiltrated by neoantigen-specific T cells. In addition, the increased number of intra-epithelial CD8^+^ T cells in these MMR-p tumors may also relate to a TGF-β transcriptional signature, since TGF-β is known to regulate tissue residency of CD8^+^ T cells by inducing the expression of integrins like αE (CD103) and α1, as well as CD69 [52]. On the other hand, TGF-β can affect T cell populations by inhibiting IL-2-dependent proliferation [53] and their cytotoxic activity, which could impair the activity of neoantigen-reactive TIL *in vivo *[54–56]. In support of this, Tauriello and colleagues have shown that the therapeutic targeting of TGF-β, in CRC models reminiscent of the CMS4 subtype, unleashes the capacity of the adaptive immune system to eradicate tumors [57]. It is likely that this suppressive environment is lost during the extraction and culturing of neoantigen-reactive T cells, thereby allowing their detection in *in vitro *systems. The relevance of TGF-β as immune suppressor has also been demonstrated in a therapeutic setting in humans: TGF-β signaling activation in tumors was associated with a lack of response upon anti-PD-L1 treatment in urothelial cancer patients [58]. Currently, several initiatives are ongoing to augment responses to immunotherapeutic interventions by concomitantly targeting the TGF-β pathway [59, 60].

Seminal work by Tran and colleagues demonstrated the feasibility of detecting neoantigen-directed T cell reactivity by TIL in gastrointestinal tumors, including CRC with moderate mutation burden (58 to 155 transcribed non-synonymous mutations) [61]. Moreover, the significant potential of neoantigen-specific T cells as therapeutic vectors in CRC has been highlighted by the successful treatment of a metastatic CRC patient by autologous cell transfer of a KRAS-mutant-reactive polyclonal T cell population [62]. Typically, the detection rate of neoantigen-specific T cell responses has been reported to range between 1 and 4% of the tested putative neoantigens [39, 61]. Therefore, *a priori*, it was unlikely that neoantigen-specific T cell responses could be detected in CRC with low mutation burden (below 50) like the ones reported in this work. Differences in methodological approaches, especially the use of RNA expression as a filter for variants to be screened, may explain such discrepancies although a greater number of research efforts are required for defining a range of detection of neoantigen-specific T cell reactivity across cancer types. Just recently, another research group demonstrated the existence of neoantigen-reactive T cells in various metastases of MMR-p gastrointestinal tumors, including CRC [45]. These data combined with ours show that neoantigen-specific T cells reside in both the primary tumor as well as metastases of CRC. Interestingly, it is known that the CMS4 subtype is overrepresented in CRC metastatic disease [63] which is in line with our observations and the fact that Parkhurst and colleagues were able to demonstrate neoantigen-specific T cell responses in the majority of tumors analyzed.

Neoantigen-specific T cell responses have also been described in other tumor types with moderate to low mutation burden like ovarian cancer [64]. Moreover, personalized vaccination strategies, consisting of autologous dendritic cells pulsed with tumor lysate, prolonged the survival of ovarian cancer patients as therapeutic responses and were shown to be largely driven against cancer neoantigens [65]. Glioblastoma is another cancer type that is traditionally viewed as non-immunogenic due to the low number of mutations that occur in this disease. Remarkably, vaccination approaches with peptides corresponding to cancer neoantigens, in a personalized setting, were shown to promote tumor-specific immune reactions in glioblastoma patients [66, 67]. Finally, a metastatic cholangiocarcinoma patient experienced disease regression and stabilization after therapeutic administration of T cell products generated from neoantigen-reactive CD4^+^ T cells that recognized one neoantigen out of 26 transcribed mutations detected in the tumor tissue [68]. The detection of neoantigen-specific T cell responses and the success of some neoantigen-targeting therapeutic approaches are highly supportive of the notion that a broader proportion of cancer patients diagnosed with different tumor types may benefit from immunotherapeutic strategies, albeit personalized approaches will be required in those solid tumors which harbor mainly neoantigens derived from passenger genes and are thus heterogenic.

While checkpoint blockade therapies are currently ineffective in MMR-p CRC, the demonstration that neoantigen-reactive T cells infiltrate these tumors supports the development of alternative immunotherapeutic approaches that could include vaccination with biomolecules corresponding to immunogenic neoantigens or adoptive cell transfer of cancer-reactive T cells. To date, most adoptive T cell transfer therapy protocols are based on the non-controlled enrichment of heterogeneous mixtures of cancer-reactive and bystander T cells that may generate therapeutic products with suboptimal anti-cancer activity. The observation that neoantigen-reactive T cells can be identified by a specific phenotype, namely through co-expression of CD39 and CD103, can support their specific enrichment for downstream cellular therapies that can include cloning of the T cell receptors on non-exhausted donor T cells [17, 40, 47]. Here, we show that neoantigen reactivity can be attributed to this CD39^+^CD103^+^ CD8^+^ T cell subset, but additional investigations are ongoing to confirm our observation. Additionally, the possibility to enrich for neoantigen-reactive CD4^+^ T cell populations requires further exploration.

When T cells fail to infiltrate or persist in cancer tissues, vaccination approaches making use of biomolecules corresponding to neoantigens might be more suitable so that priming and mobilization of neoantigen-specific T cells can occur. The adoption of this strategy may be particularly suitable for the treatment of patients with MMR-p tumors, since (1) the low neoantigen abundance allows the functional testing or therapeutic exploitation of the majority of cancer neoantigens in each patient with limited dependency on prediction algorithms and (2) these tumors are less frequently affected by immune evasion events such as defects in antigen presentation [41]. Independently of the immunotherapeutic approaches of choice, it is likely that concurrent strategies are required to provide inflammatory signals or breakdown of immune suppressive barriers for these patients. Among these, the complementary use of chemo- and radiotherapy as well as the employment of oncolytic viruses are promising approaches for the support of immunotherapies [69]. Further, and as demonstrated here, the immune infiltrate of CMS4 tumors comprises both tumor-reactive and immune-suppressive cells, resulting in a strong rationale for blocking the TGF-β pathway in tumors that exhibit features of TGF-β activation in their microenvironment to unleash pre-existing T cell reactivity.

## Conclusions

Taken together, our data demonstrate that autologous neoantigen-specific immune responses are present in patients diagnosed with MMR-p CRC of the CMS4 subtype. These findings support the adoption of specific immunotherapeutic strategies that deliver solutions for this patient group which may include neoantigen-based vaccines or enrichment of neoantigen-specific T cells for T cell therapies. The presence of neoantigen-reactive T cells in a milieu that is strongly associated with TGF-β activation also supports combinatorial strategies aimed at tackling this immune-suppressive pathway.

## Supplementary information

**Additional file 1: Table S1.** HLA class I genotypes determined by exome and transcriptome sequencing.

**Additional file 2: Table S2.** Mutant peptide sequences that were investigated for T cell reactivity.

**Additional file 3: Table S3.** Immune-regulatory gene set.

**Additional file 4: Table S4. **Antibody panels used for flow cytometric analyses.

**Additional file 5: Figure S1. **Peptide reactivity screens with TIL.

**Additional file 6: Figure S2. **Peptide reactivity screens with PBL.

**Additional file 7: Figure S3.** Peptide reactivity screens with HPLC-purified, wild type and mutant versions of putative neoantigen sequences.

**Additional file 8: Figure S4. **Relative expression level of neoantigens for samples NIC, NIC4, and NIC15 and tumor-reactivity experiment for sample NIC4.

**Additional file 9: Figure S5. **Cross-reactivity experiments to confirm patient-specific neoantigen reactivity.

**Additional file 10: Table S5.** Phenotypic analysis of the expanded TIL products.

## Data Availability

Patient’s RNA sequencing data can be retrieved from the Sequence Read Archive of NCBI via PRJNA591080.

## Abbreviations

APCs: Antigen-presenting cells
CRC: Colorectal cancer
CMS: Consensus molecular subtypes
EBV-LCL: Epstein-Barr virus–transformed lymphoblastoid B cell lines
MMR: Mismatch repair
MMR-d: Mismatch repair-deficient
MMR-p: Mismatch repair-proficient
PBL: Peripheral blood lymphocytes
PBMC: Peripheral blood mononuclear cells
Tregs: Regulatory T cells
SEB: *Staphylococcus aureus* enterotoxin B
SLP: Synthetic long peptides
SSP: Synthetic short peptides
TIL: Tumor-infiltrating lymphocytes
