# Supplementary material for: Neoantigen-specific immunity in low mutation burden colorectal cancers of the consensus molecular subtype 4
Source: Genome Med. 2019 Dec 30;11:87. doi: 10.1186/s13073-019-0697-8 (PMC6938004; doi:10.1186/s13073-019-0697-8)
Supplement: Supplementary file 5 — Additional file 5: Figure S1. Peptide reactivity screens with TIL. [file 13073_2019_697_MOESM5_ESM.pdf]

Fig. S1

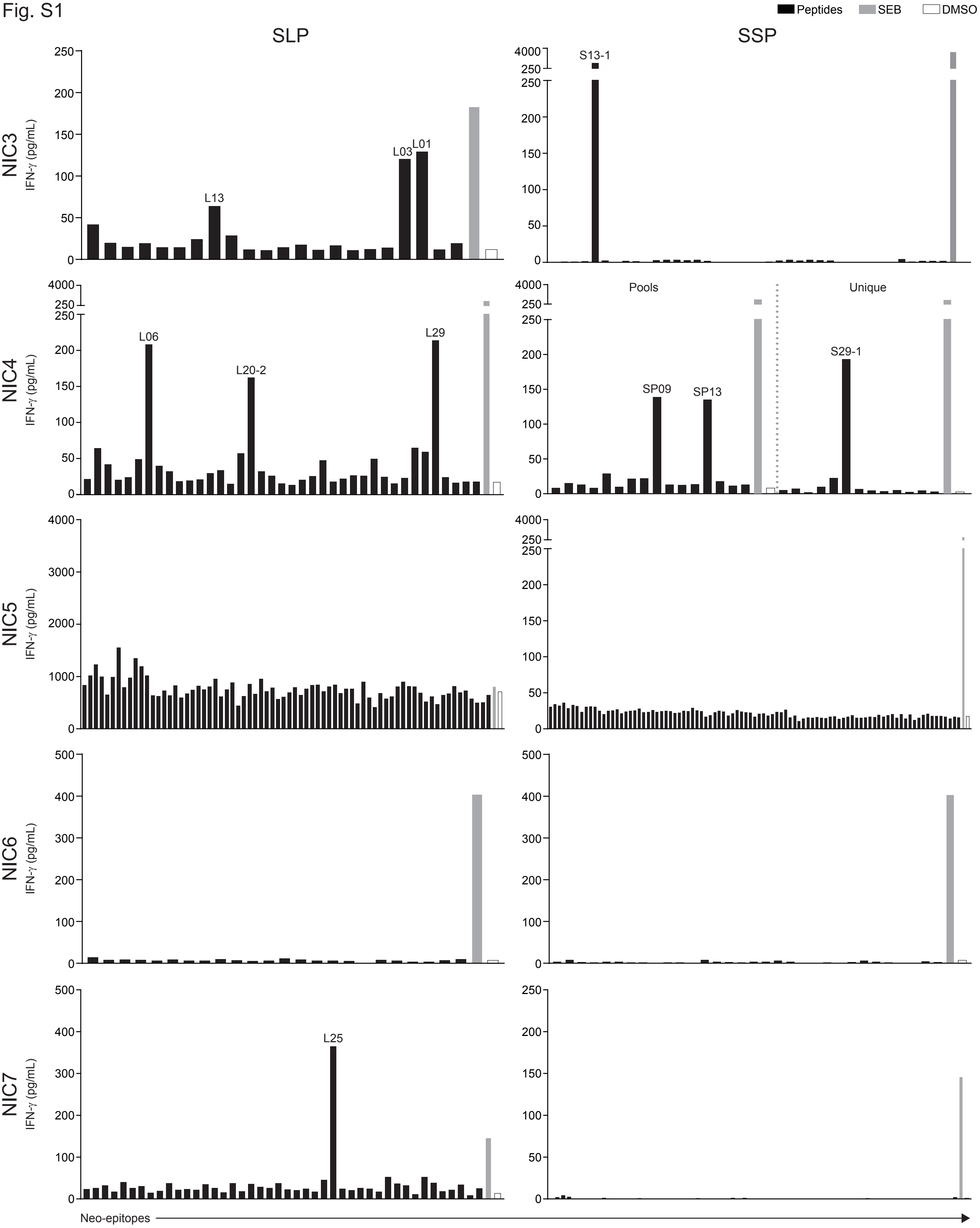

Peptides
SEB
DMSO
SLP
SSP
NIC3
NIC4
NIC5
NIC6
NIC7
IFN-γ (pg/mL)
L13
L03
L01
S13-1
L06
L20-2
L29
Pools
Unique
SP09
SP13
S29-1
L25
Neo-epitopes

Supplementary Figure 1

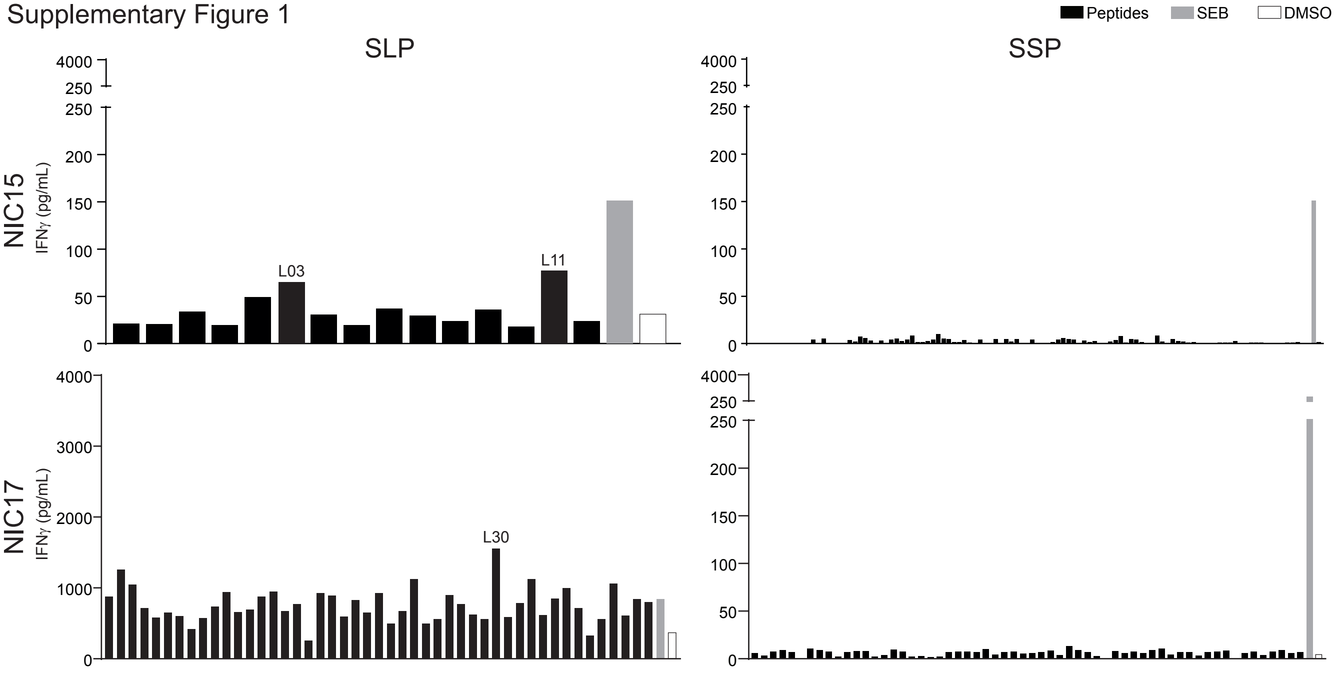

Figure S1 | Neoantigen-specific reactivity screen of the expanded TIL from NIC3-7, NIC15 and NIC17 towards SLPs and SSPs. IFN-γ production (y-axis) is shown for each neo-epitope that was tested (black bars, x-axis) and the positive (grey bar) and negative control (white bar). Peptide IDs are included for neo-epitope responses that were judged positive and selected for validation. SSPs and SLPs with the same ID number correspond to the same mutation per patient.
