# Supplementary material for: Neoantigen-specific immunity in low mutation burden colorectal cancers of the consensus molecular subtype 4
Source: Genome Med. 2019 Dec 30;11:87. doi: 10.1186/s13073-019-0697-8 (PMC6938004; doi:10.1186/s13073-019-0697-8)
Supplement: Supplementary file 6 — Additional file 6: Figure S2. Peptide reactivity screens with PBL. [file 13073_2019_697_MOESM6_ESM.pdf]

Fig. S2

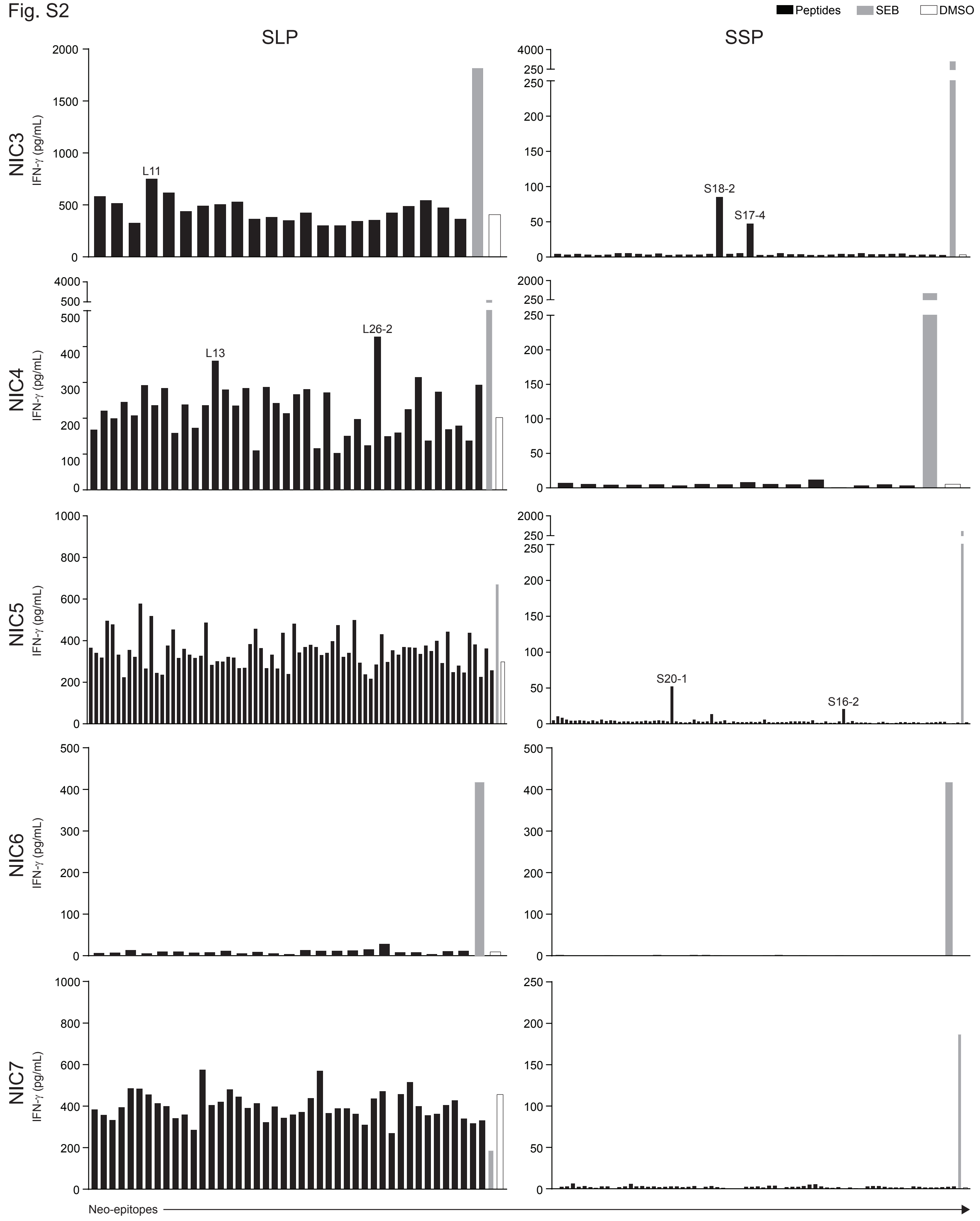
Peptides
SEB
DMSO
SLP
SSP
NIC3
NIC4
NIC5
NIC6
NIC7
IFN-γ (pg/mL)
L11
S18-2
S17-4
L13
L26-2
S20-1
S16-2
Neo-epitopes

Figure S2 | Neoantigen-specific reactivity screen of the PBL from NIC3-7 towards SLPs and SSPs. IFN-γ production (y-axis) is shown for each neo-epitope that was tested (black bars, x-axis) and the positive (grey bar) and negative control (white bar). Peptide IDs are included for neo-epitope responses that were judged positive and selected for validation. SSPs and SLPs with the same ID number correspond to the same mutation per patient.
