# Supplementary material for: Neoantigen-specific immunity in low mutation burden colorectal cancers of the consensus molecular subtype 4
Source: Genome Med. 2019 Dec 30;11:87. doi: 10.1186/s13073-019-0697-8 (PMC6938004; doi:10.1186/s13073-019-0697-8)
Supplement: Supplementary file 7 — Additional file 7: Figure S3. Peptide reactivity screens with HPLC-purified, wild type and mutant versions of putative neoantigen sequences. [file 13073_2019_697_MOESM7_ESM.pdf]

Fig. S3

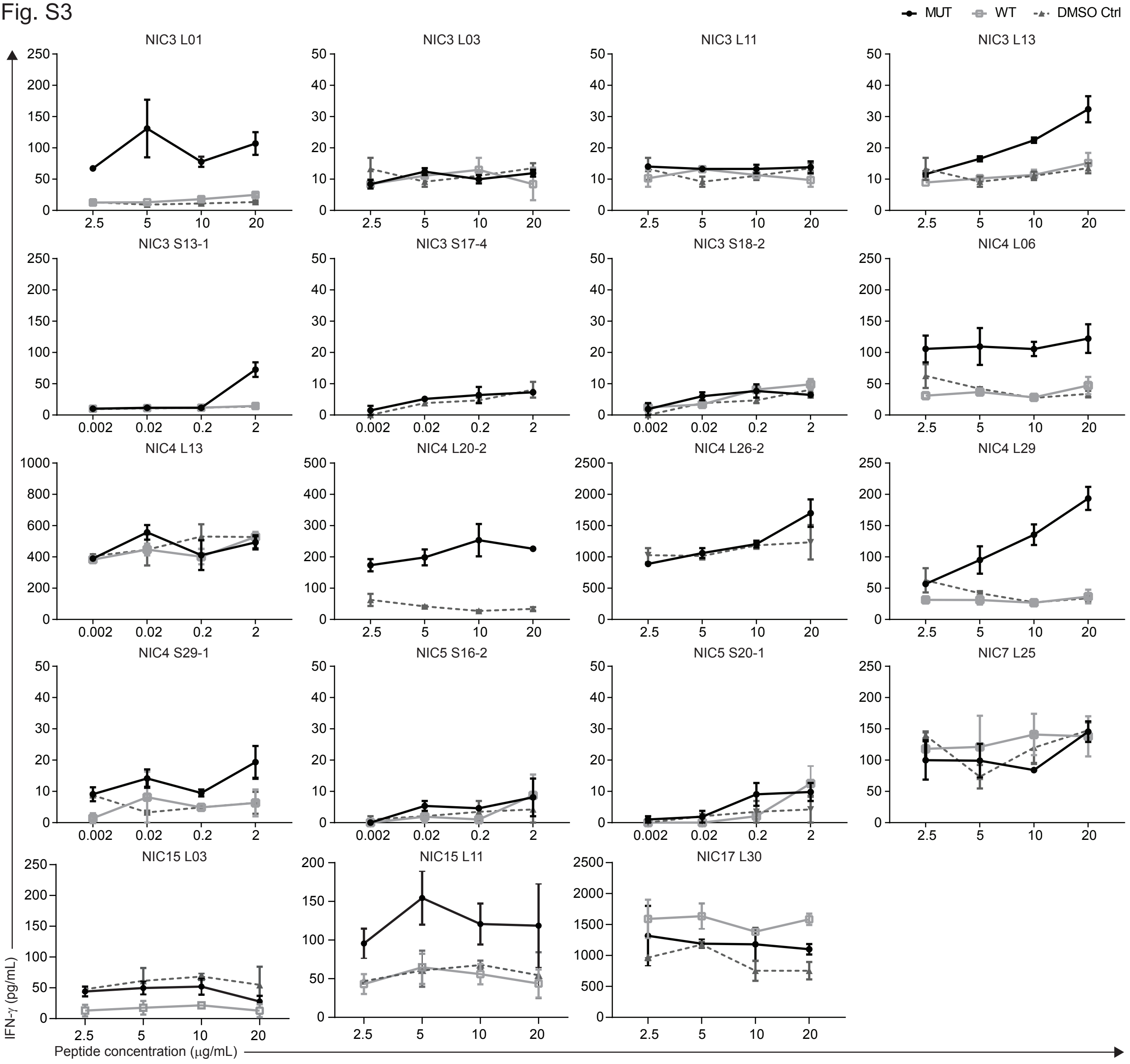

Figure S3 | Validation of the neoantigen-specific responses detected in the first screen. IFN-γ production of TIL and PBL from NIC3-7, NIC15 and NIC17 was measurement upon stimulation with mutant (black) and wild type (grey) HPLC peptides, and a DMSO control (dashed), at different peptide concentrations. The mean ± standard deviation of the biological duplicates in the same experiment are depicted.
