# Supplementary material for: Neoantigen-specific immunity in low mutation burden colorectal cancers of the consensus molecular subtype 4
Source: Genome Med. 2019 Dec 30;11:87. doi: 10.1186/s13073-019-0697-8 (PMC6938004; doi:10.1186/s13073-019-0697-8)
Supplement: Supplementary file 8 — Additional file 8: Figure S4. Relative expression level of neoantigens for samples NIC, NIC4, and NIC15 and tumor-reactivity experiment for sample NIC4. [file 13073_2019_697_MOESM8_ESM.pdf]

Fig. S4

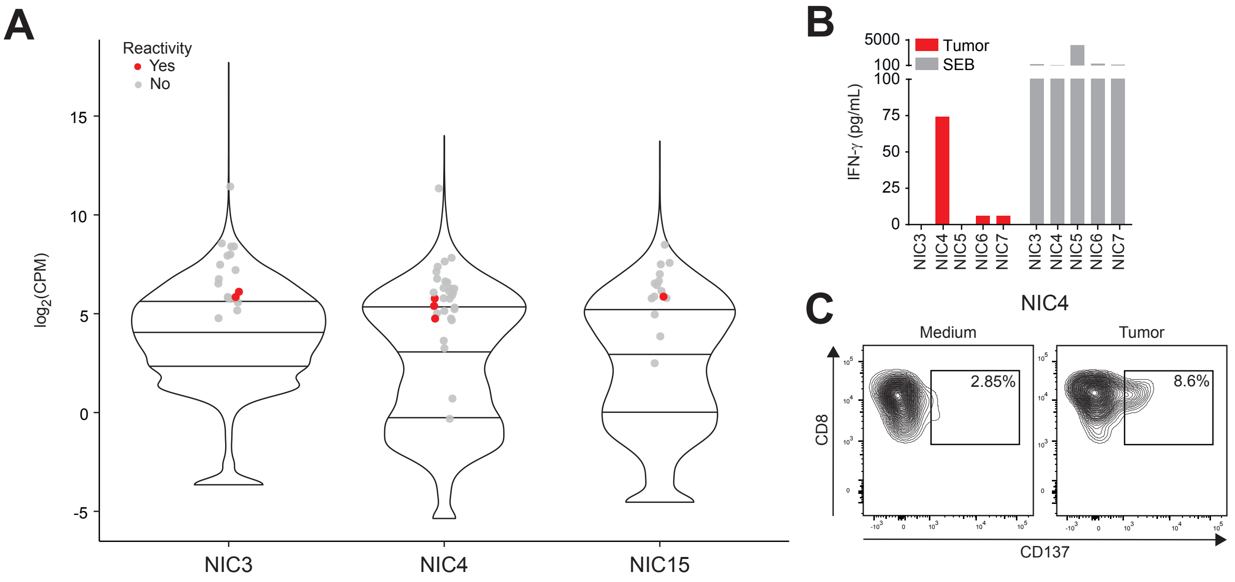

Figure S4 | a RNA expression of genes encoding somatic mutations (dots) in relation to overall gene expression level in NIC3, NIC4, and NIC15. Red dots highlight the genes to which neoantigen-specific T cell responses were observed. **b** Measurement of IFN-γ production upon co-culture of TIL with autologous tumor material (red) or SEB (positive control, grey). **c** Flow cytometric analysis of CD8+ TIL cultures derived from NIC4 shows an increase of CD137 expression upon co-culture with autologous tumor material compared to the medium control. The percentage in the gates refer to the CD137-positive fraction within the CD8+ subset.
