# Supplementary material for: Neoantigen-specific immunity in low mutation burden colorectal cancers of the consensus molecular subtype 4
Source: Genome Med. 2019 Dec 30;11:87. doi: 10.1186/s13073-019-0697-8 (PMC6938004; doi:10.1186/s13073-019-0697-8)
Supplement: Supplementary file 9 — Additional file 9: Figure S5. Cross-reactivity experiments to confirm patient-specific neoantigen reactivity. [file 13073_2019_697_MOESM9_ESM.pdf]

Fig. S5

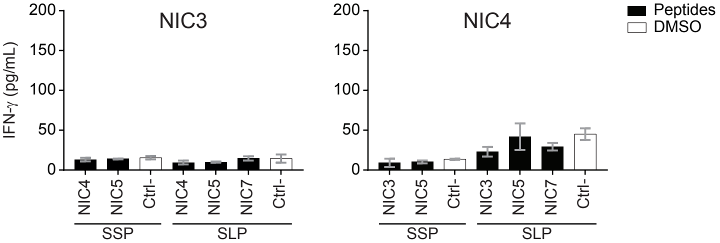

Figure S5 | Cross-reactivity of the T cells. TIL (black) were tested towards peptide pools of other patients including SLP loaded on EBV-LCL and SSP. Medium with DMSO (white) with or without EBV-LCL were taken along as negative control for the SLP and SSP, respectively. This cross-reactivity setting shows the patient-specific nature of neoantigen reactivity. The mean ± standard deviation of the biological duplicates in the same experiment are depicted.
